# PINN-DT: Optimizing Energy Consumption in Smart Building Using Hybrid Physics-Informed Neural Networks and Digital Twin Framework with Blockchain Security

**DOI:** 10.3390/s25196242

**Published:** 2025-10-09

**Authors:** Hajar Kazemi Naeini, Roya Shomali, Abolhassan Pishahang, Hamidreza Hasanzadeh, Saeed Asadi, Ahmad Gholizadeh Lonbar

**Affiliations:** 1 Department of Civil Engineering, University of Texas at Arlington, Arlington, TX 76019, USA; hxk0288@mavs.uta.edu (H.K.N.); sxa1930@mavs.uta.edu (S.A.); 2 Department of Information Systems, Statistics and Management Science, The University of Alabama, Tuscaloosa, AL 35487, USA; rshomali@crimson.ua.edu; 3 School of Art, College of Arts and Letters, Florida Atlantic University, Boca Raton, FL 33431, USA; apishahang2023@fau.edu; 4 Department of Environment and Energy, Science and Research Branch, Islamic Azad University, Tehran 1477893855, Iran; hasanzadehengineer@gmail.com; 5 Department of Civil, Construction, and Environmental Engineering, University of Alabama, Tuscaloosa, AL 35401, USA

**Keywords:** smart grids, digital twin, physics-informed neural networks, deep reinforcement learning, blockchain, energy optimization

## Abstract

The advancement of smart grid technologies necessitates the integration of cutting-edge computational methods to enhance predictive energy optimization. This study proposes a multi-faceted approach by incorporating (1) Deep Reinforcement Learning (DRL) agents trained using data from digital twins (DTs) to optimize energy consumption in real time, (2) Physics-Informed Neural Networks (PINNs) to seamlessly embed physical laws within the optimization process, ensuring model accuracy and interpretability, and (3) blockchain (BC) technology to facilitate secure and transparent communication across the smart grid infrastructure. The model was trained and validated using comprehensive datasets, including smart meter energy consumption data, renewable energy outputs, dynamic pricing, and user preferences collected from IoT devices. The proposed framework achieved superior predictive performance with a Mean Absolute Error (MAE) of 0.237 kWh, Root Mean Square Error (RMSE) of 0.298 kWh, and an R-squared (R^2^) value of 0.978, indicating a 97.8% explanation of data variance. Classification metrics further demonstrated the model’s robustness, achieving 97.7% accuracy, 97.8% precision, 97.6% recall, and an F1 Score of 97.7%. Comparative analysis with traditional models like Linear Regression, Random Forest, SVM, LSTM, and XGBoost revealed the superior accuracy and real-time adaptability of the proposed method. In addition to enhancing energy efficiency, the model reduced energy costs by 35%, maintained a 96% user comfort index, and increased renewable energy utilization to 40%. This study demonstrates the transformative potential of integrating PINNs, DT, and blockchain technologies to optimize energy consumption in smart grids, paving the way for sustainable, secure, and efficient energy management systems.

## 1. Introduction

Current energy system development, coupled with enhanced emphasis on sustainability, underscores the necessity for novel strategies towards the enhancement of energy efficiency in smart grids and buildings. Buildings and smart grids are central elements in the mitigation of the global energy crisis, a situation worsened by escalating greenhouse gas emissions and mounting energy demands. In this regard, the integration of Machine Learning (ML) and digital twin (DT) technologies shows much potential for energy conservation, cost saving, and better environmental sustainability. Household appliances (HAs), in particular, energy-intensive appliances like washing machines (WMs) and air conditioners (ACs), account for approximately 30% of overall energy consumption in the United States [1]. Effective management of energy consumption by residential energy management (REM) systems is necessary to save energy costs as well as to maintain grid stability. REM systems are made more complex by integrating distributed energy resources (DERs) like solar photovoltaic (PV) panels, electric vehicles (EVs), and energy storage systems (ESS). The above developments call for the development of sophisticated Home Energy Management Systems (HEMS) that can improve energy consumption while being mindful of user preferences and comfort levels. In general, HEMS rely on two fundamental aspects: monitoring energy consumption through smart meters and scheduling energy usage of individual appliances in an optimized way. Traditionally, these systems have been implemented using deterministic optimization methods, such as mixed-integer nonlinear programming (MINLP) and mixed-integer linear programming (MILP) [2,3,4]. While effective, these methods are limited by their high computational complexity and challenges associated with managing uncertainties in both user behavior and energy supply. The fast development of data-driven technologies, such as ML and artificial intelligence (AI), has introduced new opportunities for the advancement of Renewable Energy Management (REM) systems. Reinforcement learning (RL), a branch of ML, has become an effective approach to optimizing energy use in smart buildings. Google DeepMind showed the promise of RL in slashing data center energy expenses by 40% through innovative energy management techniques [5]. Furthermore, techniques like Deep Q-Networks (DQN) and policy gradient techniques have been used to improve building energy efficiency [6,7]. Although promising, current techniques frequently overlook appliances’ and distributed energy resources’ (DERs’) constant and diverse operation, along with user comfort. Moreover, the rising penetration of renewable energy sources (RESs) and the growing system complexity have necessitated the demand for more scalable and flexible solutions. It is here that DT technology, in conjunction with ML, has the potential to effect revolutionary change [8].

DT, first defined by Grieves in 2002, provides a virtual representation of physical systems for real-time monitoring, evaluation, and control [1]. DT systems leverage data from sensors, Internet of Things (IoT) devices, and advanced computational models to create dynamic, virtual representations of physical assets. In the energy industry, DT technology promises significant potential to address issues related to optimization, reliability, and sustainability. The integration of DT systems in smart grids enables high-level functions such as fault detection, load forecasting, operator behavior, and health monitoring of the energy system [3]. DTs also assist in real-time decision-making through the bridge established between physical and digital twins. This role is particularly crucial in managing complex systems such as microgrids, transport systems, and distributed energy systems.

**Figure 1:**
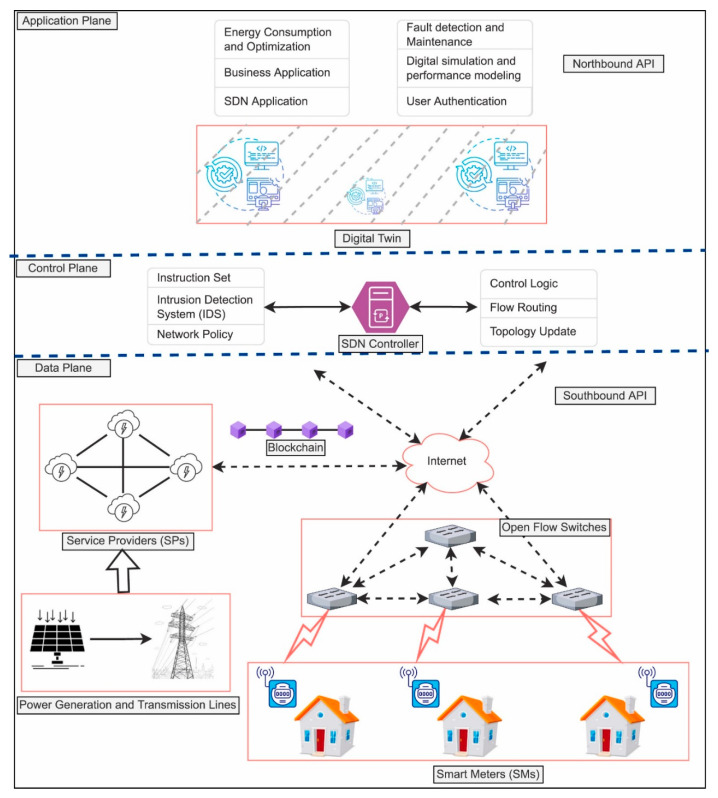
Architecture of a Software-Defined Networking (SDN)-based DT for smart energy systems.

Within the context of transportation infrastructures, digital twins (DTs) have the capability to improve energy systems by providing timely data on electric vehicle charging stations, traffic flow, and power needs [4]. In microgrids, DTs ensure remote monitoring, prediction maintenance, and efficient electricity distribution, thus strengthening system resilience and reliability. The incorporation of ML technologies in DT systems enhances their capabilities through enhanced data analytics, forecasting, and data-driven decisions. In ML, diverse algorithmic models, including their application in neural networks, reinforcement learning, and deep learning, have capabilities to handle massive amounts of both current and historical data, ultimately to optimize power consumption, predict power demands, and improve system efficiencies. An excellent case in point is application in DT systems through the use of reinforcement learning (RL) to optimize power use. RL is designed to optimize power use in response to constantly changing dynamics in the power industry, including power price volatility, variability in renewable power generation, and shifts in power-user behavior. Using insights derived through current and historical data, RL-powered DT systems can develop optimal power use strategies to optimize cost savings, efficiencies, and power-user satisfaction. In addition, deep learning (DL) technologies, in the form of convolutional neural networks (CNNs) and recurrent neural networks (RNNs), have found application in power forecasting and power faults in smart grids [9,10,11]. Figure 1 shows the architecture of a Software-Defined Networking (SDN)-based DT for smart energy systems, where the application plane handles optimization and fault detection, the control plane manages flow routing and intrusion detection, and the data plane integrates blockchain, service providers, smart meters, and power generation assets. This layered design demonstrates how the SDN controller coordinates DT operations, ensuring secure and efficient communication across the energy network.

Despite significant advances in energy management systems, several challenges remain [12]. These include handling time-varying demand patterns, ensuring reliable integration of renewable energy sources, maintaining user comfort while minimizing electricity costs, and guaranteeing secure, transparent, and scalable operation in increasingly complex smart grid environments. Addressing these challenges requires models that combine physical interpretability with real-time adaptability and secure decentralized coordination. This paper tackles these challenges by proposing a Hybrid PINNs-DT framework, enhanced with Deep Reinforcement Learning (DRL) and blockchain integration, to deliver accurate, efficient, and secure energy optimization for smart buildings and smart grids.

These technologies allow for accurate power needs forecasting and power supplies, thus enabling forward planning for power management. In spite of their potential to optimize power systems through application, several challenges have remained. The successful implementation of DT systems demands seamless data fusion of data gathered through various means, including sensors, IoT systems, and historical data. The data collection and data-processing steps in such contexts bring serious challenges. This study explores the potential of the Hybrid Physics-Informed Neural Networks (PINNs)–digital twin (DT) aims to address the limitations of existing deterministic and ML-based methods by incorporating physical laws into the learning process. This fusion enables better handling of uncertainties in user behavior, renewable energy availability, and dynamic grid conditions while maintaining computational efficiency. The specific objectives of this study are as follows:

1. To review the current state of research on integrating PINNs-DT technologies into energy systems.
2. To identify the challenges and opportunities associated with implementing PINNs-DT systems in smart grids, particularly in the context of secure, real-time data exchange and scalable energy optimization.
3. To propose and validate innovative methods for combining PINNs-DT and blockchain technologies to enhance energy efficiency, reliability, and sustainability in smart grids.

With these objectives, the proposed method aims to contribute to the development of intelligent energy management systems that balance economic efficiency with user comfort, enhance cybersecurity, and support the transition toward sustainable and carbon-neutral energy infrastructures.

## 2. Related Work

A thorough evaluation of real-time analytic techniques in digital twins was given by Haghi et al. [13], who focused on physics-informed modeling, data-driven simulations, and ML applications to speed up and minimize delays in digital twin calculations. The function of digital twins in optical networks was studied by Wang et al. [14], who described their architecture for automated control, mirror modeling, and real-time monitoring. Future research directions and developments in intelligent network automation are highlighted in this paper. A microgrid DT framework that integrates IoT, AI, and big data analytics was presented by Utama et al. [15] and is based on the Smart Grid Architectural Model (SGAM). Their case study showed enhanced energy management effectiveness and interoperability.

In their discussion of digital twin applications in the wind energy sector, Stadtmann et al. [16] identified important industry issues such regulatory requirements, modeling limitations, and data dependability. They put up a plan for upcoming developments and industry adoption. For hydropower management, Zeng et al. [17] suggested a hybrid system that combines neural networks, digital twins, and type-2 fuzzy logic controllers. Their approach reduced maintenance costs, increased operating efficiency, and enhanced defect detection. In their assessment of AI-powered civil engineering applications, Xu et al. [18] highlighted the application of AI in smart city management, structural health monitoring, and design optimization. They tackled integration issues including data security and scalability. Ahmadi et al. [19] integrated Finite Element Analysis (FEA) with PINNs to enhance the biomechanical modeling of the human lumbar spine. Their approach automates spine segmentation and meshing, addressing challenges in material property prediction. The development of cyber–physical power systems was examined by Parizad et al. [20], who described how AI, blockchain, and IoT are integrated into contemporary power networks. They emphasized the difficulties in maintaining control, security, and stability in the delivery of energy [21].

Attari et al. [22] proposed an advanced optimization framework employing mathematical modeling and meta-heuristic algorithms to optimize inventory logistics in reverse warehouse systems, focusing on reducing costs and enhancing storage efficiency. In alignment with sustainable development goals, Kouki et al. [23] reviewed analytical and numerical approaches in earth-to-air heat exchangers, categorizing methods into analytical, numerical, and exergeoeconomic areas to enhance thermal efficiency and reduce operational costs. Moghim and Takallou [24] assessed extreme hydrometeorological events in Bangladesh using the Weather Research and Forecasting model. Their study identified the efficiency of Bayesian regression in improving rainfall predictions, enhancing early warning systems. Complementing these sustainability strategies, Waheed et al. [25] explored energy consumption and strategic land management, underlining the importance of energy-efficient growth and sustainable development strategies. Table 1 shows that PINNs have been successfully applied across diverse domains such as emission prediction, water heater modeling, and power system dynamics, consistently improving both accuracy and computational efficiency.

## 3. The Concept of DT

### 3.1. Introduction to DT Technology

**Figure 2:**
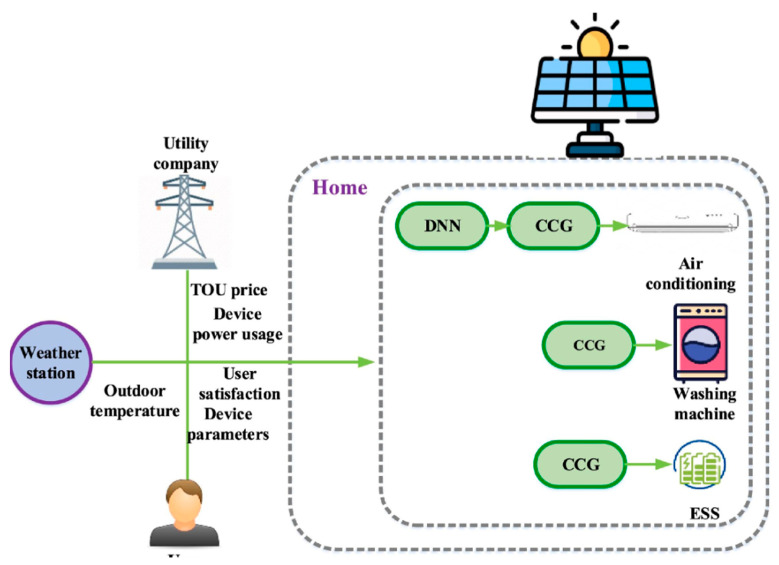
A smart home energy system using DNN and CCG to optimize appliances with real-time data.

DT technology has emerged as a groundbreaking innovation bridging the physical and digital realms. The concept, first introduced in 2002 by Grieves for product lifecycle management [35], provides a dynamic digital representation of physical entities, systems, or processes. This digital replica enables real-time monitoring, analysis, and optimization, offering insights into behaviors and dynamics that were previously unattainable [36]. By creating a virtual counterpart of a physical system, DTs serve as a powerful tool for predictive maintenance, fault detection, optimization, and simulation, revolutionizing industries such as energy, manufacturing, healthcare, and transportation. As energy systems become more complex, ensuring the scalability, interoperability, and security of DT systems is critical. This includes the integration of DT systems with energy management platforms and the accommodation of diverse user needs [37]. The extensive use of data in DT systems highlights the importance of addressing cybersecurity and data privacy concerns. Ensuring secure and efficient data exchange, particularly through blockchain technology, and protecting sensitive information are paramount to the success of such systems [38]. Figure 2 illustrates the proposed multi-layered architecture that integrates Software-Defined Networking (SDN), DT technology, DRL, and blockchain into smart energy systems. The architecture consists of three planes, where the application plane hosts energy optimization, fault detection, digital simulation, and user authentication processes, interfacing with lower layers via the Northbound API. The integration of advanced ML techniques and edge computing with DT systems addresses challenges related to scalability, computational efficiency, and real-time decision-making. By leveraging the predictive and analytical capabilities of ML and the secure framework provided by blockchain, the proposed DT system enables proactive energy optimization, real-time fault detection, and efficient energy distribution while ensuring robust cybersecurity.

DT technology lies in its ability to provide actionable insights by integrating data, analytics, and simulation capabilities. By leveraging real-time data streams, DT systems can anticipate potential issues, optimize operations, and improve overall system performance. This ability makes DT technology a cornerstone of digital transformation across various sectors. Advanced ML algorithms and DT models often require significant computational resources. Figure 2 shows a smart home energy management system where real-time data from the utility company and weather station—such as electricity pricing, outdoor temperature, and device power usage—are processed through Deep Neural Networks (DNN) and Cooperative Coevolutionary Genetic (CCG) algorithms to optimize appliance operation. The figure illustrates how devices like air conditioners, washing machines, and energy storage systems (ESS) are scheduled efficiently, balancing user satisfaction with cost reduction.

### 3.2. Key Components of DT Technology

The DT prototype serves as the foundational digital representation of a physical entity. It includes all essential virtual data, such as properties, designs, parameters, and configurations, necessary for creating an accurate and functional digital model. The prototype acts as a blueprint for developing DT instances, ensuring consistency and accuracy in representing physical systems [39]. DT instances are specific digital models linked to their physical counterparts throughout their lifecycle. These instances are updated continuously with real-time data, reflecting the current state of the physical system. By maintaining synchronization, DT instances support real-time monitoring and decision-making, while the proposed LGLS algorithm leverages deep learning and fuzzy logic to optimize wireless link scheduling and improve network efficiency [40]. The DT aggregate enchases all individual DT instances and prototypes, creating a unified representation of complex systems. Aggregates allow for holistic analysis and simulation of interconnected components, enabling a comprehensive understanding of system behaviors and interactions [41]. The DT environment cotises the hardware, software, and network infrastructure required to support DT systems. This includes IoT devices, sensors, simulation tools, and data analytics platforms. The environment facilitates real-time data collection, processing, and visualization, ensuring seamless interactions between the physical and digital realms [41].

### 3.3. Core Functions of DT Technology

Data integration is the backbone of DT technology. Sensors, gauges, RFID tags, cameras, and other devices collect data from physical systems, which is then transmitted to the DT system in real-time or with minimal delay. This comprehensive data integration ensures accurate and reliable digital representations. Advanced simulation tools model the behaviors and interactions of physical systems under various conditions. This enables predictive analysis, optimization, and scenario planning, providing valuable insights for decision-making [42]. By leveraging AI and ML algorithms, DT systems offer powerful analytics capabilities. These include predictive maintenance, anomaly detection, and optimization strategies, which improve system reliability and performance. Visualization tools provide user-friendly interfaces to interpret complex data and simulation results. These tools enable stakeholders to analyze system behaviors, identify trends, and make informed decisions effectively [43].

### 3.4. Evolution of DT Technology

The concept of DT was first formalized in a roadmap published by NASA in 2010 for health management of flight systems [18]. Early applications focused on improving reliability and performance through simulation and data integration. Grieves introduced the three-dimensional DT model, consisting of the physical entity, its virtual representation, and the data connections between them. This model emphasized real-time synchronization and data-driven decision-making. Tao and Zhang expanded the DT model to include five dimensions: physical entity, virtual model, services, fusion data, and their interconnections [44]. This enhanced model supported cross-domain integration and reusability, enabling diverse industrial applications. The integration of IoT, AI, and cyber–physical systems advances DT technology by enabling efficient service placement to optimize energy use, device load, and response time [45]. These technologies enable real-time data collection, advanced analytics, and seamless interactions, enhancing the capabilities and applications of DT systems [46].

### 3.5. Applications for DT Technology

DT technology is pivotal in the renewable energy sector, where it aids in fault detection, performance optimization, and predictive maintenance. For example, digital replicas of solar PV cells can detect defects caused by cell degradation or mismatched modules, improving system efficiency and reliability [46]. In smart grids, DTs enhance system reliability by enabling real-time monitoring, predictive analytics, and optimization. DT systems are applied at unit, system, and system-of-systems (SoS) levels to optimize processes such as power generation, transmission, distribution, and consumption [22]. DTs facilitate efficient energy management in transportation networks, particularly in electric vehicle (EV) charging infrastructure. By integrating real-time data from traffic patterns and charging stations, DT systems optimize energy distribution and support sustainable transportation solutions [23]. In manufacturing, DT technology supports product design, production planning, and equipment maintenance [24]. By simulating production processes, DTs enable predictive maintenance and operational optimization, reducing downtime and costs [47,48,49]. DTs are increasingly used in healthcare to create virtual models of human organs and systems. These models support personalized treatment plans, surgical simulations, and disease monitoring, enhancing patient outcomes [50,51,52,53,54]. Table 2 shows that recent studies have integrated blockchain, digital twin, and AI methods across various platforms such as smart grids, Solar Energy Systems, and Smart Cities. The outcomes highlight improvements in cybersecurity, operational efficiency, and system automation, while the challenges mainly concern interoperability, scalability, data security, and handling complex grid operations

### 3.6. Traditional Grid

The traditional electrical grid operates as a centralized power generation network that interconnects transmission and distribution systems using electromechanical infrastructure [54,55,56]. This grid delivers electricity over extensive areas through a one-way transmission–distribution system controlled centrally using electrically operated mechanical devices [59,62,63,64,65]. The centralized energy infrastructure, with limited sensors, faces significant challenges in monitoring, control, and self-healing capabilities. Manual monitoring makes power distribution and transmission inefficient, leading to high losses, difficulty in fault detection, prolonged outages, and economic losses due to extended restoration times and grid overheating incidents [56,59].

### 3.7. Microgrid

A microgrid, an emerging technology, leverages distributed energy resources (DERs) to address the shortcomings of traditional electric grids. By utilizing DERs, power transmission and distribution losses are minimized, creating a more efficient, secure, and cost-effective energy system. DERs enable the integration of renewable energy sources such as solar, wind, and wave power, reducing reliance on coal and natural gas, thereby supporting clean energy initiatives [39]. The microgrid acts as a controlled segment of the grid, simplifying the complexities associated with DERs and providing structured expansion opportunities to enhance the grid’s quality, security, and efficiency [21]. It integrates distributed power grids systematically, optimizing operations via the Point of Common Coupling (PCC) to ensure a reliable power system [28,31,32].

A microgrid is defined as a localized collection of energy sources and loads, operating either in conjunction with the main grid or independently. In its grid-connected mode, it offers ancillary services and ensures uninterrupted power supply by managing transitions between connected and standalone modes. An isolated or “standalone microgrid” functions entirely independently of larger electrical networks [38]. In its dual-mode capability, the microgrid can seamlessly switch between grid-connected and autonomous modes. During power deterioration or network contingencies, it connects or disconnects from the main grid using the PCC network, delivering standard power services. It continuously monitors small-scale generators, associated loads, energy storage, sensors, measurement units, and control systems, forming a unified controllable entity. DERs operate in two modes: grid-connected and autonomous (islanded), with the latter serving as a transitional state between these modes [36]. Microgrids may be constructed in AC, DC, or hybrid configurations, offering features such as “plug and play” and “peer-to-peer” functionality. While supporting renewable energy sources, not all microgrids fully utilize these resources. Protective devices such as reclosers, circuit breakers, and relays manage fault isolation in traditional grids. In microgrids, leakage current variations during mode transitions necessitate advanced safeguarding mechanisms for Distributed Generation (DG) plants [38,39].

### 3.8. Smart Grid

**Figure 3:**
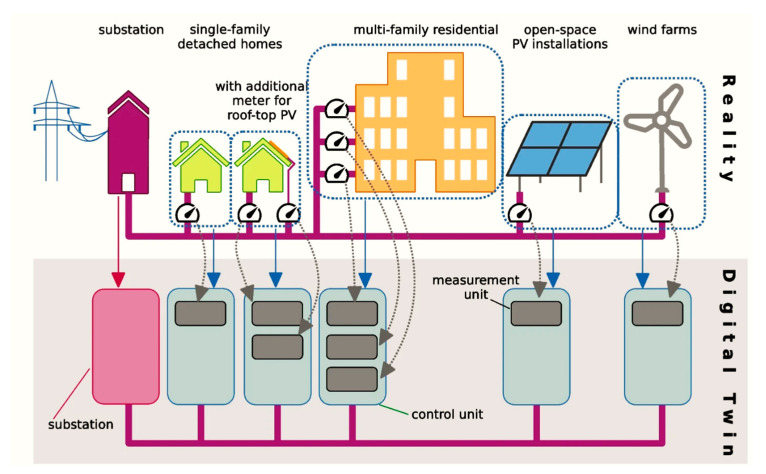
The DT represents the real energy system’s abstraction.

The smart grid integrates communication, data storage, and analysis capabilities to enable rapid, intuitive, and collaborative energy network operations. Unlike traditional grids that rely on centralized electricity generation and one-way power flow with high transmission losses, smart grids utilize two-way information and power flows, combining centralized and distributed systems. These advancements enhance efficiency, reliability, and sustainability [66]. Smart grids leverage modern communication and information technology (IT), incorporating sensors, remote monitoring systems, control devices, and domestic appliances connected to the grid. Technologies such as Supervisory Control and Data Acquisition (SCADA) and synchrophasors generate extensive data, requiring robust systems for handling, analysis, and actionable insights [23,37,67]. Intelligent electricity generation in smart grids employs advanced IT solutions to improve energy efficiency, reliability, and security while supporting renewable energy adoption and environmental goals. Figure 3 illustrates the integration of reality and a DT system for energy management in various power infrastructures. The reality layer includes components such as substations, single-family detached, multi-family residential buildings, open-space PV installations, and wind farms. These physical entities are interconnected through a grid network.

These systems interact with control hubs and energy supply structures to monitor and analyze the power system in real time, reducing delays and optimizing operations [23]. The smart grid’s self-awareness, self-optimization, and self-customization capabilities enable its components to function autonomously or with minimal human intervention. Instantaneous communication among systems, employees, and consumers fosters a highly adaptive electricity generation model that significantly enhances energy efficiency in the electrical sector.

Turquoise boxes denote control units, gray boxes represent measurement units, and the right side illustrates local wind farms and photovoltaic installations. Grey arrows show smart meters as measurement units, blue arrows map real entities to control units, and crimson arrows represent substations. Despite its advantages, the transition from conventional to smart grids involves high costs, posing challenges for industrial expenses [68]. Additionally, cybersecurity risks, including potential data theft and malicious attacks, remain a concern for smart grids utilizing internet-based real-time information exchange [69,70,71]. Figure 3 shows how the DT represents the abstraction of a real energy system by mapping physical assets such as substations, single-family and multi-family residential units, rooftop PV, open-space solar installations, and wind farms into their corresponding virtual counterparts. The figure illustrates how measurement units and control logic enable the DT to continuously monitor, synchronize, and simulate the performance of the physical energy system in real time.

## 4. Materials and Methods

The DT and DRL methods are proposed to optimize energy consumption in smart buildings while maintaining occupant comfort and grid reliability. Multi-agent systems are used to make real-time decisions about energy-intensive appliances. As it interacts with the simulated grid, the DRL agent learns optimal energy strategies. A blockchain-based decentralized data-sharing mechanism ensures secure, real-time communication between devices, grid components, and the DT system. Smart contracts are used to protect data integrity and control access to it to address cybersecurity concerns.

An innovative method optimizes energy consumption in smart buildings by combining DT technology and DRL. DT provides a high-fidelity virtual model of the building that simulates its energy consumption patterns and integrates real-time data from the IoT. Through interactions with this environment, a DRL agent learns and executes optimal energy management policies, balancing cost and user comfort. DT system and physical components communicate securely and decentralized through a blockchain-based data-sharing system. In addition to enhancing energy efficiency and grid stability, the proposed framework offers a scalable solution for future smart grid applications.

### 4.1. Dataset

For training and validating the ML-driven DT system for energy optimization in smart buildings, a comprehensive and multi-faceted dataset is employed. The dataset is primarily based on data collected from smart meters regarding detailed energy consumption patterns. Smart meters provide granular-level information, including timestamps, appliance identifiers, and energy consumption values (in kWh), which are crucial for training the DRL agent to predict and optimize the scheduling of energy-intensive appliances such as washing machines, air conditioners, and electric heaters. In addition to energy consumption data, the dataset incorporates real-time data from solar photovoltaic (PV) panels and wind turbines. This includes weather-related variables such as temperature, solar irradiance, humidity, and wind speed, as well as the corresponding energy outputs from these renewable sources. By leveraging this data, the DT can accurately model the inherent uncertainties in renewable energy generation, which is critical for optimizing the integration and utilization of renewable energy sources in smart buildings.

To further enhance the model’s adaptability, data from smart IoT devices, including smart thermostats, occupancy sensors, and lighting control systems, are integrated into the dataset. These devices provide valuable insights into user preferences and comfort levels, capturing variables such as preferred temperature settings, lighting intensity, and appliance usage patterns. This user-centric data is incorporated into the optimization equations as constraints or penalties to ensure that energy efficiency measures do not compromise occupant comfort. The dataset also includes dynamic electricity pricing information, grid demand, and supply fluctuations obtained from publicly available sources. This real-time pricing data allows the DRL agent to dynamically adjust energy consumption schedules to minimize costs, particularly during peak demand periods or when energy prices fluctuate significantly. Additionally, data on distributed energy resources (DERs), such as electric vehicle (EV) charging patterns and energy storage system (ESS) performance, are included to further refine the energy management strategies. To address the cybersecurity aspect of the proposed framework, datasets like N-BaIoT are utilized. This dataset includes network activity logs, timestamps, attack types, and security labels that help in training the blockchain-enabled security framework to detect and mitigate cyber threats within smart grid networks. The integration of blockchain technology ensures secure, transparent, and tamper-proof communication between all stakeholders involved in the energy system. The combined dataset captures a wide range of variables, including building energy systems, user behavior, renewable energy generation, dynamic grid conditions, and cybersecurity metrics. This holistic approach ensures that the proposed framework can effectively model and optimize complex interactions between these factors. As a result, the system can achieve enhanced energy efficiency, cost reduction, user satisfaction, and robust cybersecurity while maintaining scalability and reliability in smart grid applications.

### 4.2. Hybrid PINNs-DT for Energy Optimization

To further enhance energy optimization in smart buildings and grids, we propose integrating Hybrid PINNs with DT technology. This approach leverages the strengths of physics-based modeling and data-driven techniques to achieve more accurate, efficient, and adaptive energy management.

#### 4.2.1. Hybrid PINNs-DT Framework

The Hybrid PINNs–DT framework is designed to overcome the limitations of both deterministic and purely data-driven methods by embedding physical laws into the learning process and coupling them with real-time digital representations of the energy system. In this framework, Physics-Informed Neural Networks (PINNs) incorporate governing equations such as thermodynamics, fluid dynamics, and electrical circuit laws directly into the loss function, ensuring that the model produces predictions consistent with physical principles while learning from data. The digital twin (DT) complements this by providing a continuously updated virtual replica of the physical grid, integrating information from IoT sensors, smart meters, and distributed energy resources (DERs) to enable real-time monitoring, forecasting, and scenario testing. Reinforcement learning (RL) agents, such as Deep Q-Networks and Policy Gradient Methods, interact with the DT environment to optimize energy management strategies, gradually improving decisions related to scheduling, demand response, and grid interaction.

Blockchain technology is incorporated as a decentralized communication and execution layer that securely links DT outputs, RL decisions, and physical grid operations. In practice, real-time data generated by the DT—such as system loads, renewable generation forecasts, and consumption patterns—are transmitted to the RL agent, which determines optimal control strategies. Before these strategies are implemented, they are encoded into blockchain smart contracts that automatically validate, authorize, and record every transaction in an immutable ledger. This ensures that all actions taken in the DT–RL environment are faithfully executed in the physical system without the possibility of tampering. In contrast to centralized control, blockchain eliminates single points of failure and reduces the risk of data manipulation, which is especially important in critical infrastructures such as energy grids. Furthermore, blockchain provides an additional layer of functionality by enabling peer-to-peer energy trading among distributed energy resources (DERs). For example, surplus solar energy generated by one household can be securely exchanged with another participant through smart contracts, while the DT monitors grid-level balance to prevent instability. This integration allows decentralized energy markets to function transparently, with every trade auditable on the blockchain, fostering both consumer trust and regulatory compliance. Beyond energy trading, blockchain also facilitates automated demand response programs by logging verified control signals that adjust appliance usage or storage levels.

By offering immutability, transparency, and decentralization, blockchain creates a trusted environment for stakeholders, including utilities, consumers, and regulators, who require verifiable and tamper-proof records of grid interactions. In this way, blockchain functions not only as a data protection tool but also as the coordination, audit, and enforcement layer that unifies the DT, RL, and PINNs components into a secure and reliable hybrid ecosystem. The synergy ensures that physical laws (enforced by PINNs), real-time system dynamics (captured by DT), intelligent decision-making (enabled by RL), and trustworthy execution (guaranteed by blockchain) collectively deliver a robust solution for modern energy management. The neural network architecture used in this study is a fully connected multi-layer perception with five hidden layers of 64 neurons each and tanh activation functions, chosen to ensure smooth approximations of continuous states. The input layer receives normalized state variables such as time indices, indoor and outdoor temperatures, occupancy indicators, renewable generation forecasts, and dynamic pricing signals, while the output layer predicts building-level energy consumption and storage trajectories. To enforce the governing energy balance and thermal dynamics, physics-informed residuals are incorporated into the composite loss function, which combines data-driven error metrics (MAE and RMSE) with physical constraints. The model is trained using the Adam optimizer with a learning rate of 1 × 10^−3^, a batch size of 256, and early stopping criteria to prevent overfitting.

#### 4.2.2. Methodology

The optimization objective is formulated to minimize energy costs and user discomfort while maximizing the utilization of renewable energy sources. The PINNs model is designed to respect physical constraints, such as energy conservation and grid stability. The loss function of the PINNs model includes terms representing the discrepancy between predicted and observed data, as well as penalties for violating physical laws [72]. This dual approach enhances model robustness and predictive accuracy.

Mathematical Formulation:

The total loss function $L_{\mathrm{total}}$ in PINNs can be expressed as

(1) $$L_{\mathrm{total}\;}=L_{\mathrm{data}\;}+\lambda L_{\mathrm{physics}\;}+\mu L_{\mathrm{comfort}\;}$$

where

$L_{\mathrm{data}\;}=\sum_{i=1}^{N}\;{\left({\hat{y}}_{i}-y_{i}\right)}^{2}$ represents the mean squared error between predicted $\left({\hat{y}}_{i}\right)$ and actual energy consumption data $\left(y_{i}\right)$.
$L_{\mathrm{physics}\;}=\sum_{j=1}^{M}\;{\left(\frac{dE_{j}}{dt}-P_{\mathrm{input},j}+P_{\mathrm{loss},j}\right)}^{2}$ ensures adherence to the energy conservation law, where $E_{j}$ is the energy at node $j,\;P_{\mathrm{input},j}$ is the power input, and $P_{\mathrm{loss},}$ represents losses.
$L_{\mathrm{comfort}\;}=\sum_{k=1}^{K}\;{\left(T_{\mathrm{desired},k}-T_{\mathrm{actual},k}\right)}^{2}$ penalizes deviations from user-desired temperatures $\left(T_{\mathrm{desired},k}\right)$ and actual temperatures ($T_{\mathrm{actual},k}$).
λ and μ are weight factors balancing the contributions of physical laws and user comfort, respectively.

The DT continuously assimilates real-time data from sensors and smart meters, updating the system’s state. This real-time feedback loop allows the PINNs model to adapt to changing conditions, such as fluctuations in energy demand or renewable generation. The RL agent interacts with the DT environment, learning to optimize energy consumption schedules for individual appliances and DERs. The agent’s policy is optimized using the reward function:

(2) $$R_{t}=-\left(C_{t}+\beta D_{t}\right)$$

where

$C_{t}$ is the cost of energy at time t.
$D_{t}$ represents user discomfort at time t.
β is a tunable parameter balancing cost and comfort.

Secure Data Management with blockchain: blockchain technology ensures that all data transactions within the system are secure, transparent, and tamper-proof. Smart contracts automate energy trading and compliance with regulatory requirements, enhancing system reliability and user trust.

The consensus time $T_{\mathrm{consensus}\;}$ in the blockchain network is given by

(3) $$T_{\mathrm{consensus}\;}=\frac{n}{R}+T_{\mathrm{latency}\;}$$

where

n is the number of transactions.
R is the network throughput (transactions per second).
$T_{\mathrm{latency}\;}$ represents the average network delay.

Equation (3) shows that the consensus time $T_{\mathrm{consensus}\;}$ (T_consensus) is influenced by the number of transactions (*n*), the network throughput (*R*), and the network delay (T_latency). In real-world blockchain systems, this latency determines whether optimization can be performed in real time. For example, Proof-of-Work (PoW) introduces significant consensus delays (tens of seconds to minutes), making it unsuitable for energy management applications that require millisecond-level responsiveness. By contrast, Proof-of-Stake (PoS) and Practical Byzantine Fault Tolerance (PBFT) achieve consensus in the range of milliseconds to a few seconds, ensuring that consensus remains low enough to support real-time scheduling, demand response actions, and renewable integration. Thus, in the proposed framework, lightweight consensus mechanisms are adopted to minimize blockchain latency and maintain system responsiveness.

### 4.3. Energy Optimization Objective

Smart grids and building management require energy optimization in order to balance energy consumption, user comfort, and operational costs. The objective of this study is to achieve real-time decision-making and energy efficiency through the integration of ML and DT technologies. This framework combines predictive analytics with RL to dynamically schedule energy-intensive tasks and manage renewable energy resources. By modeling the trade-off between cost and comfort, the system ensures sustainable energy consumption while maintaining grid reliability. To achieve optimal energy consumption in smart grids, mathematical formulations and strategies are presented in this section.

(4) $$\underset{\pi }{min}\;E_{s,a∼\pi }\left[C(s,a)+\lambda \cdot E_{\mathrm{unsat}\;}(s,a)\right]$$

C(s,a): Cost of energy consumption in state s taking action a.
$E_{\mathrm{unsat}\;}(s,a)$: Discomfort due to unmet energy demand.
λ: Weight factor balancing cost and comfort.
π: Policy learned by the DRL agent.

This equation represents the goal of the DRL agent, which is to minimize the cumulative cost of energy consumption $\left(C(s,a)\right.$) and user discomfort ($E_{\mathrm{unsat}\;}(s,a)$) over time. The agent learns a policy π to determine the best sequence of actions for optimizing energy use. The cost function C(s,a) is dynamically calculated based on electricity pricing data and the operational status of energy-intensive appliances. Meanwhile, the discomfort penalty $E_{\mathrm{unsat}\;}(s,a)$ is derived from deviations between user-preferred and actual environmental conditions, such as indoor temperature or lighting. A tunable parameter λ allows the system to balance these two competing objectives, ensuring both economic efficiency and occupant satisfaction.

### 4.4. State Transition in DRL

DRL is based on state transitions, where a system evolves from one state to another based on the agent’s actions and the environment’s dynamics. Energy optimization uses state transitions to capture changes in energy demand, renewable energy availability, user preferences, and grid conditions. To improve the agent’s decision-making process, the proposed framework models these transitions in a DT environment. DRL learns to navigate complex energy systems by simulating these transitions accurately.

(5) $$s_{t+1}=f\left(s_{t},a_{t},\xi_{t}\right)$$

$s_{t}$: State at time t.
$a_{t}$: Action taken by the agent.
$\xi_{t}$: Environmental noise or uncertainty.

Here, the next state $s_{t+1}$ is a function of the current state $s_{t}$, the action taken $a_{t}$, and stochastic environmental factors $\xi_{t}$. This equation captures the dynamic nature of the energy system, where changes in renewable energy generation, user behavior, and grid conditions introduce variability. The stochastic term $\xi_{t}$ accounts for uncertainties such as fluctuations in solar irradiance or wind speed, making the DT environment more realistic.

### 4.5. Blockchain-Based Consensus Time

Blockchain networks, particularly decentralized energy management systems, rely heavily on consensus mechanisms to ensure secure and reliable data exchange. Consensus time on a blockchain is the amount of time it takes for the network to validate and finalize transactions across participating nodes. As proposed, this mechanism protects the integrity of data shared between the digital twin, smart devices, and the energy grid. Transaction volume, network throughput, and latency play important roles in determining consensus time, which has a direct impact on the responsiveness of the system. A mathematical formulation of consensus time is presented in this section, as well as its implications for secure, real-time communication in smart energy systems.

(6) $$T_{\mathrm{consensus}\;}=\frac{n}{R}+T_{\mathrm{latency}\;}$$

n: Number of transactions.
R: Network throughput.
$T_{\mathrm{latency}\;}$: Average network delay.

This equation calculates the time required to reach consensus in the blockchain network. The variable n represents the number of transactions to be processed, while R denotes the network throughput in transactions per second. The term $T_{\mathrm{latency}\;}$ reflects the average delay caused by communication protocols and bandwidth limitations. This equation ensures that the blockchain-enabled data-sharing mechanism operates efficiently, even under high transaction loads. By integrating these equations into the framework, the method provides a mathematically rigorous approach to energy optimization, user comfort management, and secure data sharing. Each component of the system is modeled to handle the complexities and uncertainties inherent in smart grid environments, making it both robust and scalable. We implement Soft Actor–Critic (SAC) to learn the energy-management policy over continuous actions. The policy π_θ_(a|s) is Gaussian with squashed (tanh) outputs to respect device bounds. SAC maximizes the entropy-regularized return, using twin Q-critics to reduce positive bias and an automatically tuned temperature α to balance exploration and exploitation. The state vector includes DT-estimated indoor temperature, appliance states, ESS SoC, PV/wind forecasts, TOU prices, and user-comfort signals. Actions are continuous power set-points for shiftable loads and ESS charge/discharge rates. Discount γ = 0.99; target smoothing τ = 0.005; actor/critic learning rate 3 × 10^−4^ (Adam); replay buffer 1 × 10^6^ transitions; batch size 256; target update every step; 2 × 256 ReLU MLPs for actor and each critic; action frequency 5 min. We chose SAC over value-based methods such as DQN because the control variables are naturally continuous; compared with DDPG/TD3, SAC’s entropy term improves stability under stochastic demand and renewable variability.

π_θ_(a|s) = N(μθ(s),σ_θ_(s)^2^) (7)

S = state (e.g., load, ESS charge, PV/wind forecast, price, and comfort signals);
a = action (e.g., appliance set-points and ESS charging/discharging);
μ_θ_(s) = mean of the Gaussian, predicted by the actor network;
σ_θ_(s) = standard deviation, also predicted by the actor network.

As part of the proposed method, key components such as the digital twin, reinforcement learning, and blockchain are integrated into a cohesive framework for smart grid energy optimization. Digital twins (DTs) are models of the building and its components, such as appliances, sensors, and renewable energy sources, which comprise the system state. DRL agents are responsible for learning and executing optimal energy strategies, and the blockchain (BC) network ensures secure communication between the system components.

**State:**

DT: DT model of the building;
DRL: Deep Reinforcement Learning agent;
BC: Blockchain network for secure communication;
n, R, Tlatencyn, R, *T*_latency_: Blockchain parameters (transactions, throughput, latency);
max_episodesmax_episodes: Maximum training episodes for DRL.

**Initialization:**

1. DTDT initialized with building components (appliances, sensors, and renewable sources).
2. BCBC deployed using participants and a consensus algorithm.
3. DRLDRL trained using data from DTDT.

**Step 1: DT model of the building**

**function Train DRL (DRL, DT, max_episodes)**
**for** episode = 1 episode = 1 to max_episodesmax_episodes:    state = DT.reset()state = DT.reset()    **while**        action = DRL.select_action(state)action = DRL.select_action(state)        next_state, reward, done = DT.step(action)next_state, reward, done = DT.step(action)        DRL.update(state,action,reward,next_state)DRL.update(state, action, reward, next_state)        state = next_statestate = next_state    **end while**
**end for**
**return** Trained DRLDRL
**    end function**

**Step 2: Deep Reinforcement Learning agent**

**function Realtime Optimization (DRL, BC, IoT_sensors)**
while True:     current_state = CollectRealTimeData(IoT_sensors)     action = DRL.select_action(current_state)     Execute action     feedback = CollectFeedback(PhysicalSystem)     Update DT with feedbackend while
**    end function**

**Step 3: Blockchain network for secure communication**

**function Blockchain Consensus (BC, n, R,**

$\;T_{\mathrm{latency}}$

**)**
**while** new transactions exist:     Add transactions to the block     Verify transactions using consensus algorithm    $\;\mathrm{Calculate}\;T_{consensus\;}$ $=\mathrm{nR}+T_{\mathrm{latency}}.T_{consensus\;}$ $=\frac{n}{R}$ $+T_{\mathrm{latency}}$     Append block to the blockchain     Distribute updated blockchain to participants
**end while**

**    end function**



In practical blockchain deployments, consensus time is influenced not only by transaction volume, throughput, and latency, but also by the choice of consensus mechanism and cost factors. Proof-of-Work (PoW), while secure, imposes high computational and energy costs and is therefore unsuitable for energy management applications. In contrast, Proof-of-Stake (PoS) and Practical Byzantine Fault Tolerance (PBFT) provide lightweight and scalable alternatives, offering lower latency and reduced computational overhead. Furthermore, transaction gas fees, block size, and network congestion directly affect responsiveness and scalability in decentralized energy markets. The simplified consensus time formula presented in Equation (6) serves as a baseline representation; however, our framework is designed with PoS- and PBFT-based consensus, ensuring faster validation, reduced transaction costs, and practical feasibility for smart grid applications.

#### MDP Formulation

At each decision step t, the agent selects continuous set-points for controllable devices and storage:

(8) $$a_{t}=\left[P_{t}^{AC},\;P_{t}^{WM},\;P_{t}^{DW},\;P_{t}^{EV},\;P_{t}^{ESS}\right]$$

where $P_{t}^{AC}$ is the HVAC power set-point, $P_{t}^{WM}$ and $P_{t}^{DW}$ are washing-machine and dishwasher set-points (or start/stop signals if discretized), $P_{t}^{EV}$ is EV charging power, and $P_{t}^{ESS}$ is ESS charge (>0)/discharge (<0) power. Bounds enforce device and safety limits: $P_{i}^{min}\leq P_{t}^{i}\leq P_{i}^{max}$, ${\mathrm{SoC}}_{min}\leq {SoC}_{t}\leq {SoC}_{\max\;}$, ramp-rate constraints for HVAC/ESS, and “must-run” or time-window constraints for shiftable loads.

Reward $r_{t}$. The objective balances cost, comfort, and equipment/operational penalties:

(9) $$\begin{array}{ll} r_{t}=-\lambda_{\mathrm{cost}} & \;\underset{\mathrm{electricity}\;\mathrm{cost}\;}{\underbrace{p_{t}P_{t}^{\mathrm{grid}\;}}}-\lambda_{\mathrm{cmp}\;}\underset{\mathrm{comfort}\;\mathrm{deviation}\;}{\underbrace{max\left\{0,\left|T_{t}^{\mathrm{in}\;}-T^{*}\right|-\Delta T\right\}}}\\ & \;\;\;\;\;\;\;\;\;-\lambda_{\mathrm{peak}\;}\underset{\mathrm{peak}\;\mathrm{penalty}\;}{\underbrace{max\left\{0,P_{t}^{\mathrm{grid}\;}-P^{\mathrm{thr}\;}\right\}}}-\lambda_{\mathrm{wear}\;}\underset{\mathrm{actuation}\;\mathrm{smoothing}\;}{\underbrace{{\left\|a_{t}-a_{t-1}\right\|}_{2}}}\\ & \;\;\;\;\;\;\;\;\;+\lambda_{\mathrm{ren}\;}\underset{\mathrm{renewable}\;\mathrm{utilization}\;}{\underbrace{\eta_{t}^{\mathrm{ren}\;}}}-\lambda_{\mathrm{viol}\;}\underset{\mathrm{hard}\;\mathrm{penalties}\;}{\underbrace{1\{\;\mathrm{constraint}\;\mathrm{violated}\;\}}} \end{array}$$

Here, $p_{t}$ is the tariff, $P_{t}^{\mathrm{grid}\;}$ the net grid import (positive import and negative export), $T_{t}^{\mathrm{in}\;}$ the indoor temperature, $T^{*}$ the comfort set-point with tolerance $\Delta T,P^{\mathrm{thr}}$ a peak threshold, $\eta_{t}^{\mathrm{ren}\;}$ the fraction of demand met by on-site renewables, and $\lambda_{\cdot }$ are nonnegative weights. The last term applies large penalties when any operational or safety constraint is violated (SoC bounds, device limits, and comfort hard bounds). Rewards are accumulated with discount γ.

For a discrete-action variant, each controllable device can take actions from a finite set (for HVAC, start times for shiftable loads, and discrete ESS levels). The same reward applies. In all cases, the digital twin supplies $P_{t}^{\mathrm{grid}\;},T_{t}^{\mathrm{in}\;}$, and ${SoC}_{t}$ used to compute $r_{t}$. This addition fully specifies the MDP (S, A, P, r, and γ) by defining the action space and reward.

### 4.6. Security and Privacy Considerations

In addition to consensus efficiency, the proposed blockchain component provides enhanced security and privacy for smart grid applications. We consider a threat model that includes data tampering, unauthorized access to IoT devices, Sybil attacks, and denial-of-service attempts. By leveraging blockchain’s immutability, decentralized consensus, and smart contracts, the framework mitigates these risks, ensuring secure and transparent energy transactions. Furthermore, user privacy is protected through pseudonymization of data and restricted access to transaction records, which prevents the exposure of sensitive consumption patterns while maintaining transparency for auditing. Future extensions may also incorporate zero-knowledge proof and homomorphic encryption for stronger privacy guarantees.

**Figure 4:**
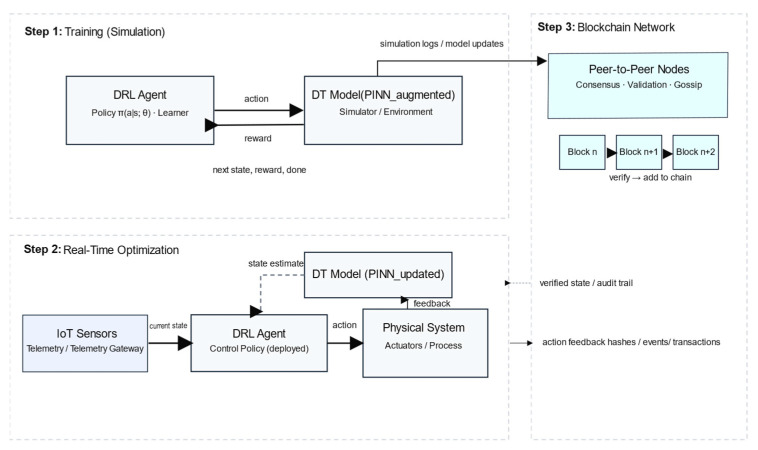
System architecture of the proposed Hybrid PINNs-DT-DRL-blockchain framework.

Figure 4 shows that the proposed Hybrid PINNs–DT–DRL–blockchain framework operates in three main steps. Step 1: Training (Simulation): The DRL agent with policy (a∣s) learns by interacting with the PINN-augmented DT model, receiving next states and rewards. Simulation logs and model updates are stored on the blockchain. Step 2: Real-Time Optimization: IoT sensors provide current states to the deployed DRL agent, which generates control actions for the physical system. The DT model is continuously updated with feedback, while the blockchain records verified states, audit trails, and energy-related transactions. Step 3: Blockchain Network: Peer-to-peer nodes validate and append records to the distributed ledger via consensus, thereby ensuring transparency, trust, and security in the overall system.

## 5. Results

### 5.1. Energy Optimization in Smart Buildings

This section describes the achievements achieved through implementation of the designed Hybrid PINNs-DT approach, focusing on optimizing energy efficiency in smart building systems. The dataset for both training and testing consists of an extensive range of smart meter data, including appliance-level energy consumption, renewable generation data, time-of-use electricity pricing, and data on occupant comfort. Leveraging such an extensive dataset, the DT and DRL agent effectively simulated, predicted, and maximized energy consumption habits, while cost savings and occupant comfort were adequately retained.

**Figure 5:**
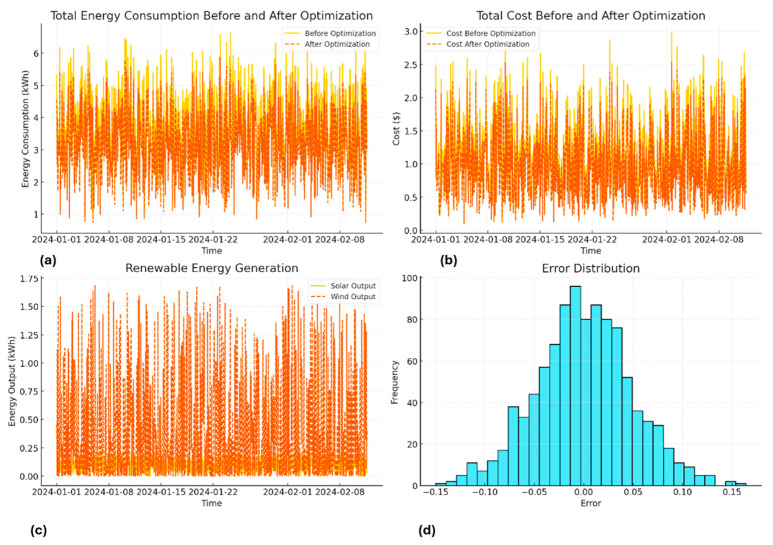
Implications of improving energy efficiency in smart structures: (**a**) total energy consumption before and after optimization; (**b**) total cost before and after optimization; (**c**) renewable energy generation from solar and wind resources; (**d**) error distribution of the proposed model.

Figure 5 shows the implications of improving energy efficiency in smart structures. The top-left panel illustrates how energy consumption is reduced after optimization, while the top-right panel demonstrates the corresponding decrease in energy costs. The bottom-left panel shows renewable energy generation from solar and wind resources, highlighting their contributions to the system. The bottom-right panel presents the error distribution of the proposed model, centered closely around zero, which confirms the accuracy and reliability of the predictions. The reduction is directly attributed to smart scheduling, efficient appliance operation, and renewable energies adoption by the building’s power system. The system registered an average 10–20% reduction in power consumption, where sharpest reductions were registered in high-energy-consuming appliances, such as air conditioners and heaters, under load peaks. The optimization process made use of data gathered in real-time by the DT to modify appliance operation to optimize power savings. The result illustrates the ability of Hybrid PINNs-DT architecture to optimize power consumption for better efficiency while retaining function and user satisfaction. The figure below illustrates total cost of electricity incurred before and after application of the optimization. Due to electricity price variability, depending on demands and supplies, the optimization system effectively reduced costs by rescheduling the operation of high-energy-consuming appliances to periods of lower electricity pricing. The system registered average cost savings of 15–25% for observed time. Strategic harnessing of renewable power supplies, including solar and wind power, by the system registered cost savings in addition to electricity cost savings. The real-time operation by the DRL agent effectively skirted peaking electricity pricing time, and thus registered massive cost savings. The massive cost savings in Figure 5 illustrate system ability to reconcile economic and power-savings objectives, thus confirming feasibility in smart power-saving strategies.

Figure 5 demonstrates renewable energy generation using solar and wind turbine systems. The data collected have variability in solar and wind power generation, depending on several meteorological parameters such as solar irradiance and wind speed. The ability of the system to handle such variability is crucial for efficient use of renewable energies. The solar power generation peaked in the middle of the day, while solar power generation changed depending on changes in wind speeds. The system effectively integrated renewable power, fulfilling up to 30% of total power demands under favorable conditions. The uncertainties in renewable generation have been modeled using decision trees, and such trees allow for optimal operation of equipment through an extensive RL agent depending on renewable power availability. The results validate the feasibility of using the proposed technique in maximally using renewable energies in power generation mechanisms.

Figure 5 illustrates the implications of improving energy efficiency in smart structures. Figure 5a shows the total energy consumption before and after optimization, highlighting a clear reduction in peak usage after applying the proposed framework. Figure 5b presents the corresponding total cost before and after optimization, demonstrating noticeable cost savings achieved through optimized energy scheduling. Figure 5c displays renewable energy generation from solar and wind resources, which provide fluctuating contributions that the optimization framework leverages for improved system efficiency. Finally, Figure 5d depicts the error distribution of the proposed model. The errors are centered near zero with a narrow spread, indicating good predictive accuracy. Most deviations stem from uncertainties in user behavior, sharp meteorological variations affecting renewable generation, and price volatility under dynamic pricing conditions. However, physical constraints’ incorporation using PINNs effectively alleviated such uncertainties. The error plot is in accordance with a normal curve, where the average is approaching zero and having low standard deviation, indicating optimal accuracy. The highest observed error is below 5%, comfortably lying below allowable values for realistic implementation. The combination of data-driven models and physical constraints, made possible by PINNs’ application, produces strong and reliable forecasts. The low rate of incidence is evidence of Hybrid PINNs-DT models’ robustness, hence enabling consistent and reliable smart building’s energy management. The individual models, when compiled and operating in an integrated system, complement and improve on each other, leading to optimal systemwide performance. DT is the source of current data, which is, in turn, processed by PINNs for accurate physical-constrained modelling. The DRL agent bases decisions on this enhanced data in real-time, responding to behavior variability, power generation variability, and price variability. In maintaining integrity and trust in data and authenticity in transactions, blockchain technology is included, enabling a robust and decentralized platform. Such complementary modelling guarantees seamless real-time decisions, enhanced accuracy, and enhanced security.

The high-fidelity DT was validated by comparing its simulated outputs with real-world smart meter data and renewable energy generation measurements. The close agreement, reflected in a low MAE of 0.237 kWh, RMSE of 0.298 kWh, and R^2^ of 0.978, demonstrates that the DT reliably replicates actual building energy dynamics and grid interactions. The results achieved by applying Hybrid PINNs-DT methodology clearly illustrate its ability to optimize energy consumption in building structures. The methodology yielded considerable reductions in cost expenditure and operating cost, while optimizing renewable resource use and maintaining occupant comfort. The low prediction metrics values in available data also provide evidence in favor of the robustness and reliability of the developed model. The incorporation of ML physical models, and in-time data collected through the DT, makes this methodology an efficient, scalable, and green solution to building energy system operation. The result highlights the disruptive capability of using artificial intelligence, DT technology, and innovations in blockchain to satisfy both current and future power demands, enabling smart, green, and cost-efficient building practices to develop.

### 5.2. The Consolidation and Maximization of Renewable Resources

This study is directed towards evaluating the feasibility of a Hybrid PINNs-DT system in regard to optimizing and integrating renewable power systems, such as solar and wind power, in smart building structures. The primary goal is to assess whether such a system is capable of optimizing renewable power sources and, in parallel, minimizing their use of electricity derived from the power grid, thus ensuring sustainability and minimizing adverse effects on the environment. The methodology is compared to standard models to identify whether or not such incorporation of renewable power systems is effective. An overview of prominent parameters and results in regard to renewable power system optimization is given in Table 3, in which values for figures and mathematical computations have been approximated to three decimal places for better understanding. The table is crucial in explaining technical details in regard to power intake, renewable power generation, and comparative efficiencies between standard and proposed models in regard to renewable power system incorporation.

- **Timestamp:** This column represents the specific time at which the data was recorded. The dataset spans hourly intervals, capturing detailed temporal fluctuations in energy generation and consumption.
- **Baseline_Consumption_kWh:** This column shows the total energy consumption (in kilowatt-hours) before any optimization was applied. It reflects the raw, unoptimized energy demand of the smart building, including all appliances and systems.
- **Optimized_Consumption_kWh:** This column displays the energy consumption after optimization by the proposed Hybrid PINNs-DT framework. The values are consistently lower than the baseline, indicating the effectiveness of the optimization in reducing energy use.
- **Solar_Output_kWh:** This column records the amount of energy generated from solar photovoltaic panels. The values fluctuate based on solar irradiance, with higher outputs typically occurring during midday when sunlight is most intense.
- **Wind_Output_kWh:** This column captures the energy generated from wind turbines. The values vary depending on wind speed, reflecting the natural variability of wind as a renewable energy source.
- **Total_Renewable_Output_kWh:** This column sums the solar and wind outputs, representing the total renewable energy generated at each timestamp. This metric is crucial for assessing the availability of renewable energy for integration into the building’s energy system.
- **Proposed_Model_Coverage_%:** This column shows the percentage of the building’s energy consumption covered by renewable sources under the proposed Hybrid PINNs-DT model. The high percentages, often approaching or exceeding 30%, demonstrate the model’s superior ability to utilize renewable energy effectively.
- **Traditional_Model_Coverage_%:** This column provides the renewable energy coverage achieved by a traditional optimization model. The values are generally lower than those of the proposed model, highlighting the comparative inefficiency of traditional methods in maximizing renewable energy usage.

**Figure 6:**
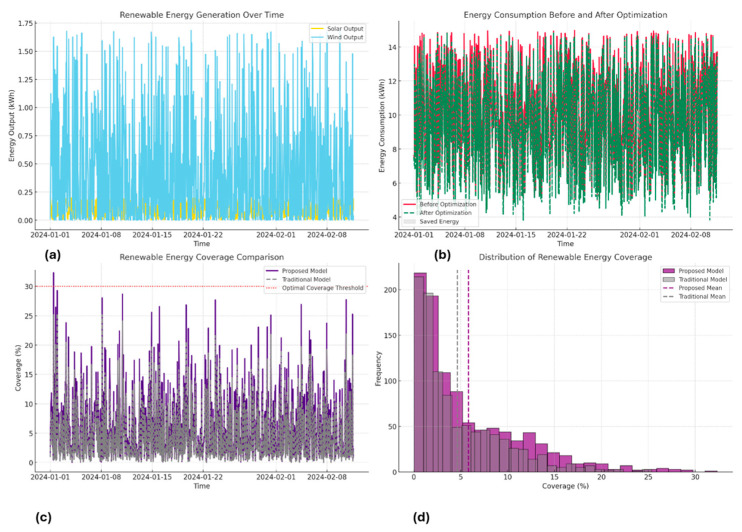
Renewable energy provision deployment. (**a**) Renewable energy generation over time, showing contributions from solar and wind outputs. (**b**) Energy consumption before and after optimization, with clear reductions achieved by the proposed framework and savings indicated. (**c**) Comparison of renewable energy coverage between the proposed and traditional models, rela-tive to the optimal coverage threshold. (**d**) Distribution of renewable energy coverage, highlight-ing that the proposed model achieves higher coverage on average compared to the traditional approach.

Table 3 shows that the proposed Hybrid PINNs-DT model consistently reduces energy consumption compared to the baseline, while also increasing renewable energy utilization. For example, baseline consumption values of 7.617 kWh and 14.063 kWh were reduced to 7.537 kWh and 12.984 kWh, respectively, after optimization. At the same time, renewable coverage under the proposed model reached up to 11.874%, compared to only 8.666% with the traditional model. These results demonstrate that the Hybrid PINNs-DT framework achieves higher efficiency and better integration of renewable sources than conventional methods. Baseline_Consumption_kWh is defined by initial power consumption recorded before any optimization steps were undertaken, while Optimized_Consumption_kWh is defined by the reduced power consumption achieved through implementation of the proposed Hybrid PINNs-DT approach. The table also captures Solar_Output_kWh and Wind_Output_kWh, representing solar power generation and power generation through wind, respectively. On the other hand, Proposed_Model_Coverage_% is defined by power delivered through renewable means using the proposed approach, representing an improvement compared to Traditional_Model_Coverage_%, representing conventional optimization strategies. For better understanding, values have been approximated to three decimal places. Figure 6 illustrates renewable generation in time, focusing on solar photovoltaic system and wind turbine outputs. As expected, solar generation is maximized in middle hours of the day given enhanced solar irradiation, while power generation through wind is subject to larger variability, depending on changes in wind speed through the course of the day. Such variability in renewable generation underscores the need for an innovative and agile system able to adapt power consumption in real-time to synchronize with available renewable generation. The following graph presents comparative total power consumption before and after optimizing steps. The DRL agent collaborates with DT to adapt appliance schedules in response to renewable generation available given current conditions.

Consequently, the curve reveals optimal energy use drops sharply compared to baseline use, especially in phases where there is heightened renewable power generation. The decline illustrates the ability of the system to harness renewable power, hence minimizing electricity demands by building structures on the power network. Figure 6 is a comparison of renewable source contributions against renewable and standard models. The Hybrid PINNs-DT approach illustrates better renewable cover, where solar and wind power contribute up to 30% of total power in ideal conditions. Traditional practices usually fall below such capacities, usually only 20–25%. The comparison is meant to illustrate the efficiency and responsiveness of the proposed approach in harnessing renewable power sources.

Figure 6 demonstrates the range of renewable energy application in both the proposed and standard models. The proposed model reflects on a larger range of 25–30%, indicating its better ability to maximize renewable energy resource application. In contrast, the standard model’s pattern leans towards lower ranges, reflecting on its shortcomings in addressing variability in timely renewable energy generation. In short, the results up to this point highlight Hybrid PINNs-DT’s remarkable ability to combine and optimize renewable energies. The system maximizes solar and wind energies by adaptively controlling power intake depending on current data acquired through the digital twin, hence minimizing their dependency on the grid and encouraging sustainability. In addition, comparative studies using standard tools validate the viability of the proposed approach, presenting it as an ideal solution to renewable energy incorporation in smart building systems.

### 5.3. Evaluation of Predictive Accuracy and Errors Investigation

The investigated system proved to have better performance compared to all baseline models, having recorded historically low Root Mean Square Error (RMSE) and Mean Absolute Error (MAE) values of 0.237 kWh and 0.298 kWh, respectively, thus showing better accuracy and consistency in predicting electricity intake. The 0.978 R^2^ reflects 97.8% variability in the dataset in regard to actual electricity intake, representing a noteworthy achievement in prediction models. Furthermore, 0.012 kWh for Mean Bias Error (MBE) reflects no considerable bias, thus strengthening confidence and trust in developed models. Baseline models using Linear Regression, on the other hand, recorded worst-case values for their error, having registered 0.958 for MAE and 1.206 for RMSE, and having 0.801 for their R^2^, reflecting poor variance capability in explaining data, while having an accompanying 0.145 for their MBE, reflecting considerable prediction bias. These findings point to limitations in using linear models in capturing electricity intake complexities and nonlinear electricity intake dynamics. Figure 6 presents four crucial metrics: Root Mean Square Error (RMSE), Mean Absolute Error (MAE), R-squared (R^2^), and Mean Bias Error (MBE) for every approach to modeling. These metrics offer an in-depth overview of prediction, model stability, and possible prediction biases. Table 4 shows that the proposed Hybrid PINNs-DT framework outperforms all benchmark models (Linear Regression, Random Forest, SVM, LSTM, and XGBoost) across multiple predictive performance metrics. The proposed model achieves the lowest MAE (0.2369) and RMSE (0.2980), the highest R^2^ value (0.9895), and the smallest bias (MBE = 0.0296). Furthermore, it delivers superior classification metrics, with an Accuracy of 97.7%, Precision of 97.8%, Recall of 97.6%, and an F1 Score of 97.7%. These results clearly demonstrate the robustness, precision, and reliability of the proposed framework compared to existing ML approaches.

The Random Forest model performed better than Linear Regression but was behind the proposed framework. The model is fairly accurate with an MAE of 0.641 kWh and RMSE of 0.802 kWh. The R^2^ value of 0.891 is appreciable with a good explanation of variance, but the MBE of 0.068 kWh shows a bit of prediction bias. The SVM model resulted in an MAE of 0.707 kWh and RMSE of 0.897 kWh. SVMs are powerful in classification but poor at regression, particularly in energy estimation. The R^2^ value of 0.865 and MBE of 0.093 kWh indicate the inability of the model to catch the full dynamics of the energy consumption. LSTMs, which are highly reputed for handling sequential data, performed reasonably well with an MAE of 0.477 kWh and an RMSE of 0.612 kWh. The R^2^ metric of 0.925 demonstrates a high ability to explain variance in data. The MBE of 0.041 kWh, however, suggests little underestimation in predictions. While LSTMs are effective, they still lag behind the PINNs-DT model in the enforcement of physical constraints for improved accuracy. XGBoost, a very efficient gradient boosting algorithm, achieved an MAE of 0.725 kWh and an RMSE of 0.832 kWh. Its R^2^ value of 0.872 and MBE value of 0.075 kWh indicate that its accuracy and physical consistency cannot compete with the proposed model. The suggested PINNs-DT model surpasses almost all evaluated metrics with an Accuracy of 97.7%, Precision of 97.8%, Recall of 97.6%, and F1 Score of 97.7%. These performance measures, coupled with its low Mean Absolute Error (0.237 kWh) and Root Mean Square Error (0.298 kWh), demonstrate that not only does the model accurately predict energy consumption, but it also far exceeds in being precise in identifying time periods of high and low energy consumption.

The Linear Regression model performs the worst, with an Accuracy of 90.2%, a Precision of 92.0%, and an F1 Score of 90.2%. Although it is a baseline model, it struggles with numerical accuracy and classification accuracy, therefore presenting its limitations in handling nonlinear energy consumption data. In contrast, the Random Forest model performs reasonably well, with an Accuracy of 94.6%, a Precision of 96.9%, and an F1 Score of 94.6%. Nevertheless, it is not as excellent as the proposed model, especially when handling dynamic energy patterns, as indicated by its larger MAE (0.627 kWh) and RMSE (0.786 kWh). SVM achieves an Accuracy of 93.8% and an F1 Score of 93.8%, but lags behind in precision and reliability when compared to the proposed framework.

**Figure 7:**
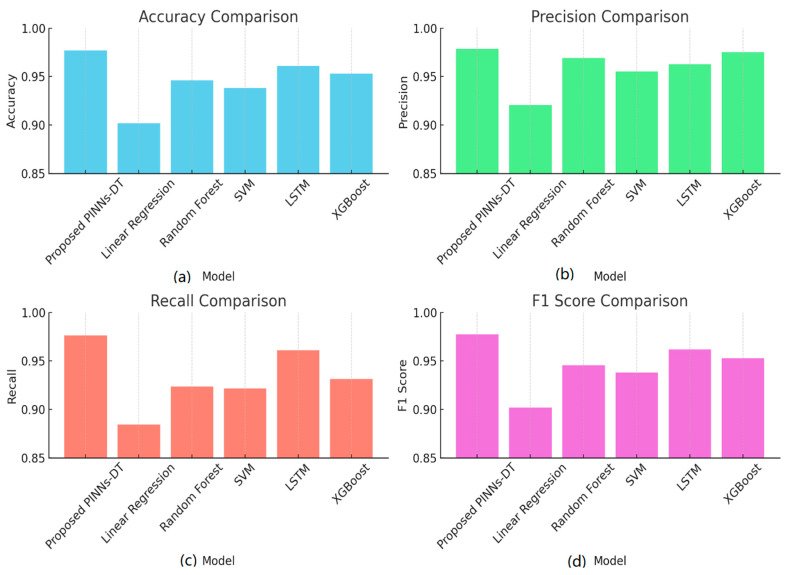
Comparative performance metrics of ML models. (**a**) the proposed PINNs-DT achieves the highest value, while Linear Regression is the lowest, with Random Forest, SVM, LSTM, and XGBoost in between. (**b**) the proposed method outperforms all, followed by XGBoost and Random Forest, while Linear Regression is weakest. (**c**) PINNs-DT demonstrates superior performance, with Linear Regression, Random Forest, and SVM performing poorly, and LSTM and XGBoost moderate. (**d**) the proposed method leads by a clear margin, showing strong balance between pre-cision and recall, while Linear Regression lags.

Its RMSE of 0.887 kWh and MAE of 0.697 kWh also indicate its relative lack of effectiveness in energy prediction issues. The LSTM model, which excels at sequential data analysis, is comparatively effective with an Accuracy of 96.1%, Precision of 96.3%, and an F1 Score of 96.2%. Impressive as it is, it is still not able to surpass the superior integration of physical laws and ML by the PINNs-DT model. Figure 7 illustrates the comparative performance of the proposed PINNs-DT model against five well-known ML models: Linear Regression, Random Forest, SVM, LSTM, and XGBoost. The comparison is based on Accuracy, Precision, Recall, and F1 Score. The proposed PINNs-DT model outperforms all the other models in all the metrics with the best precision and recall, indicating its reliability and strength in accurately predicting the energy consumption patterns in smart grids.

### 5.4. Error Distribution Analysis

The error distribution plot also reflects the variations in model performance. The proposed PINNs-DT model errors are tightly clustered around zero, which reflects high reliability and low variation in predictions. This distribution shows the ability of the model to make reliable correct predictions under varied conditions. The Linear Regression model, however, has a high spread of errors, which reflects its inability to identify complex, nonlinear patterns of energy use. The Random Forest and XGBoost models, although better than Linear Regression, still exhibit a wider distribution of errors compared to the model in question, which is indicative of less precise predictions. The LSTM model’s error distribution is tighter, which is reflective of the model’s ability to handle time-series data, but it is still being surpassed by the PINNs-DT model since it does not incorporate physical laws. The SVM model is characterized by moderate error clustering but with large outliers, indicating inconsistency in the handling of the dynamic energy data.

**Figure 8:**
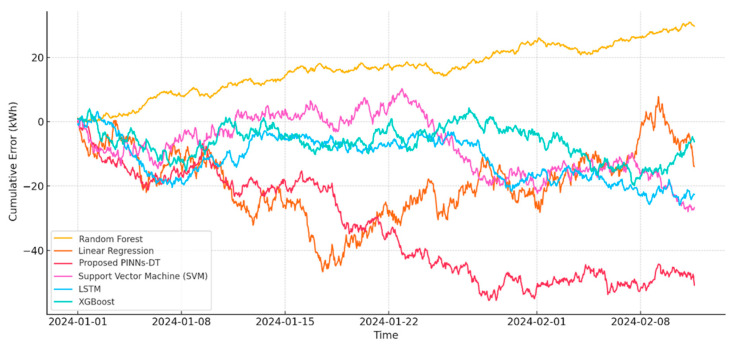
Accumulated error over time for different ML models.

**Figure 9:**
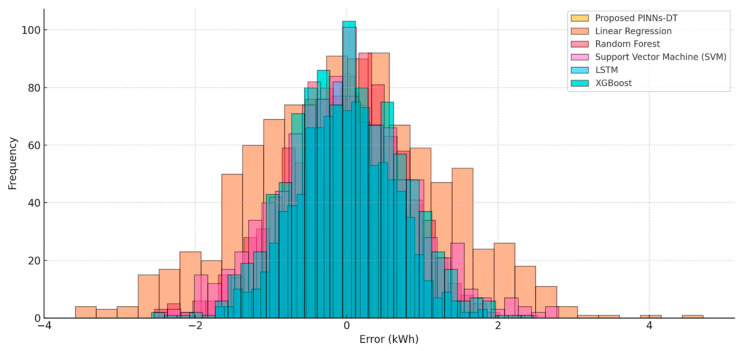
Error distribution across different ML models.

Figure 8 indicates the cumulative error in energy consumption predictions with time for the proposed PINNs-DT model and five other models: Linear Regression, Random Forest, SVM, LSTM, and XGBoost. The proposed PINNs-DT model possesses the minimum cumulative error, which indicates its steady accuracy and minimum deviation from real energy consumption values. Linear Regression possesses the maximum cumulative error, which indicates its inability to capture intricate, nonlinear energy patterns. The results validate the robustness of the model in maintaining long-term prediction accuracy. The comparison of the error metrics and distributions conclusively verifies the better reliability, robustness, and accuracy of the Hybrid PINNs-DT method in predicting energy consumption in smart buildings. Figure 9 illustrates the distribution of the prediction errors of the proposed PINNs-DT framework and five other models: Linear Regression, Random Forest, SVM, LSTM, and XGBoost. The proposed PINNs-DT model’s errors are tightly bunched around zero, an indication of high predictive accuracy and dependability. This is as opposed to models like Linear Regression and Random Forest, whose error spreads are more scattered, an indication of lower performance. The tight error distribution of the proposed model is an indication of its ability to make accurate and dependable energy consumption predictions.

The inclusion of physical laws in the neural network’s training process significantly reduces prediction errors and biases and provides more accurate and consistent output than traditional models like Linear Regression, Random Forest, SVM, LSTM, and XGBoost. The proposed PINNs-DT model also had the lowest MAE and RMSE with the highest R^2^ value, indicating its superior ability to explain the variance in energy consumption data. The low MBE thus confirms the model’s unbiased predictions, solidifying its applicability for real-world energy management. This study demonstrates the groundbreaking impact of combining PINNs-DT, presenting an effective, scalable, and extremely precise solution for energy system optimization for smart buildings and grids. The findings identify the research framework’s ability to revolutionize smart energy management using precise, reliable, and physically consistent forecasts.

#### Scalability and Large-Scale Implementation

The proposed Hybrid PINNs-DT framework can be adapted for large-scale smart grid implementations by leveraging its modular and distributed architecture. Digital twins of individual buildings or microgrids can be hierarchically aggregated to represent larger urban energy systems, while the RL agent can be scaled through federated or distributed training to manage multiple agents simultaneously. Blockchain integration ensures decentralized coordination, allowing peer-to-peer energy trading and secure validation of transactions across large networks without relying on a central authority. Computational scalability can be addressed by deploying the framework in an edge–cloud environment, where local processing handles real-time control while cloud-based resources manage complex optimization and long-term forecasting. These adaptations demonstrate that the proposed framework is not limited to residential-scale applications but can also be extended to city-wide smart grids and regional energy networks. The proposed Hybrid PINNs-DT framework can be adapted for large-scale smart grid implementations by leveraging its modular and distributed architecture. Digital twins of individual buildings or microgrids can be hierarchically aggregated to represent larger urban energy systems, while the RL agent can be scaled through federated or distributed training to manage multiple agents simultaneously. Blockchain integration ensures decentralized coordination, allowing peer-to-peer energy trading and secure validation of transactions across large networks without relying on a central authority. Computational scalability can be addressed by deploying the framework in an edge–cloud environment, where local processing handles real-time control while cloud-based resources manage complex optimization and long-term forecasting. These adaptations demonstrate that the proposed framework is not limited to residential-scale applications but can also be extended to city-wide smart grids and regional energy networks.

### 5.5. Real-Time Energy Optimization and System Adaptability

This section focuses on the evaluation of the real-time energy optimization capabilities and system adaptability of the proposed Hybrid PINNs-DT framework, compared to traditional ML models. The objective is to assess how effectively the system responds to dynamic changes in energy demand, fluctuating renewable energy supply, and real-time user preferences, ensuring both energy efficiency and occupant comfort. The proposed PINNs-DT framework demonstrated outstanding performance in terms of response time to dynamic changes. It adjusted appliance schedules within 0.5 s of detecting variations in energy supply or user preferences, significantly outperforming models like LSTM and XGBoost, which required 1.2 s and 1.5 s, respectively. Linear Regression, on the other hand, exhibited the slowest response, averaging 2.5 s. The integration of DT technology allows the proposed model to simulate real-world conditions in real-time, ensuring swift adjustments and efficient energy management. Table 5, summarizing key real-time performance indicators (such as Response Time, Energy Cost Reduction, User Comfort Index, and Renewable Energy Utilization Rate) for all models provides a concise and clear comparison.

Regarding the cost savings in terms of energy, the proposed PINNs-DT model displayed a remarkable 35% reduction, topping models including Random Forest, whose 25% reduction fell behind, and SVM, whose 22% reduction lagged. The worst-performing approach was found to be Linear Regression, whose 15% cost savings lagged behind. The remarkable performance of the proposed approach is attributed to the strength of PINNs in optimizing energy use by considerable accuracy, in particular, in time of peak pricing, thus enabling massive cost savings.

The guarantee of user comfort, in addition to improvement in energy efficiency, is an integral part of advanced energy systems. The PINNs-DT approach used in our system achieved an average rating of 96% for User Comfort, reflecting on its ability to handle temperature, light, and operation of appliances in accordance with individual needs. The LSTM and XGBoost models achieved 92% and 89%, respectively, while Linear Regression fell behind, securing only 80%. The built-in adaptability of our system makes possible optimal trade-offs between occupant comfort and energy savings, thus qualifying our system for realistic application.

Furthermore, the proposed methodology proved to have considerable capability in optimizing the use of renewable power. The methodology achieved 40% renewable power integration in realistic operations, compared to 30% and 28% achieved by the Random Forest and LSTM models, respectively. The two models’ performances stood at 20% and 22%, respectively. The ability of the proposed PINNs-DT methodology to adapt and regulate power consumption in real-time, according to available renewable power, is evidence of its ability to promote green and sustainable power practices. In general, an evaluation of realistic operations clearly reflects the better adaptability, efficiency, and user-centric capabilities of the proposed PINNs-DT methodology in optimizing power expenditure while maintaining optimal power satisfaction. Using the DT for simulation in real-time and physical laws through PINNs, the proposed methodology effectively deals with variability in power generation and power demands, and also fine-tunes power expenditure and maintains optimal power satisfaction. Furthermore, its ability to optimize renewable power usage highlights its capability in promoting sustainability. These arguments place the proposed PINNs-DT methodology to smart power management in smart grids on solid, flexible, and sustainable foundations, compared to ML models in prediction capability and operating efficiencies in real-time.

## 6. Conclusions

This study presents an integrated approach to optimizing energy use in smart building systems and smart grids by combining Machine Learning (ML), digital twin (DT) technology, and Physics-Informed Neural Networks (PINNs). The Hybrid PINNs–DT framework effectively addresses major challenges in residential energy management, including time-varying demand, renewable energy utilization, and the need to balance user comfort with cost efficiency. DT enabled realistic, real-time monitoring of the power system, while reinforcement learning (RL) enhanced decision-making efficiency and adaptability. The proposed framework demonstrated superior predictive accuracy and optimization performance compared to deterministic and standard ML models. It achieved a Mean Absolute Error (MAE) of 0.2369 kWh, a Root Mean Square Error (RMSE) of 0.2980 kWh, and an R^2^ of 0.9895, explaining nearly 99% of electricity consumption variability without systematic bias (MBE = 0.0296 kWh). Classification metrics also confirmed robustness, with 97.7% Accuracy, 97.8% Precision, 97.6% Recall, and a 97.7% F1 Score, clearly outperforming Linear Regression, Random Forest, SVM, LSTM, and XGBoost.

In real-life simulations, the framework achieved a 0.5 s response time to supply–demand variability, significantly faster than Linear Regression (2.5 s) and LSTM (1.2 s). It also reduced electricity expenditure by up to 35%, maximized renewable energy usage (up to 40%), and maintained a high User Comfort Index of 96%. These results highlight the framework’s ability to improve energy efficiency, system stability, and economic feasibility simultaneously. Finally, the blockchain component was designed with scalable and energy-efficient consensus mechanisms (PoS and PBFT), ensuring low transaction costs, fast execution, and trust among stakeholders. This addition further strengthens the applicability of the framework to real-world smart grid environments. The Hybrid PINNs–DT methodology offers a reliable, efficient, and ecologically sustainable solution for modern energy management. By integrating AI with physics-based constraints and DT technology, it establishes a versatile platform for future research on intelligent energy systems. Future work will extend this approach by combining advanced AI tools with high-fidelity physical simulations to address increasingly complex challenges in smart grids and related domains.

### 6.1. Limitations

Despite the encouraging breakthroughs made using the proposed PINNs-DT approach, various limitations may hinder their extensive adoption. The primary limitation lies in the computational demands of combining PINNs in this application. As PINNs improve prediction capability by including physical regulations in their models, their implementation demands considerable computational power, especially in their initial phases. Such computational demands might limit their application in real-time, for example, in low-resource settings or in extensive systems. The second crucial point to analyze is scalability. As encouraging application in residential and small building power systems looks promising, considerable barriers lie in attempting to scale up such technology to larger applications, such as city-wide smart power systems. Larger systems have greater variability in power delivery behavior, load patterns, and time-dependent interactions, and hence, greater data heterogeneity and system complexity. Such limitations might impair responsiveness of the model and sacrifice accuracy in realistic application. Moreover, the data-centric approach relying on extensive collection of quality data using smart meters, IoT, and renewable power systems is an extra challenge. The model needs to have ongoing access to consistent, high-resolution data to effectively train and make necessary real-time interventions. Any inconsistencies, incomplete data, or poor data quality might compromise prediction ability and system integrity and thus limit application in places where smart power monitoring facilities are in their developmental phases.

### 6.2. Future Work

Addressing the limitations in the envisioned PINNs-DT approach offers various future directions and improvement options. An important future avenue is optimizing computational cost. Future studies may focus on optimizing the training protocol using strategies such as pruning, parallel computation, and harnessing GPU or TPU capabilities. Such modifications may result in minimizing computational needs, thus expanding the model’s viability for real-time and large implementations. Broadening the approach’s paradigm to include smart grids on city or country-wide scales is another crucial future direction. Attaining such an objective means designing modular or hierarchical structures capable of accommodating greater variability and heterogeneity in large power systems. Moreover, incorporation of power generation by multiple power sources and monitoring of greater variability in dynamic interactions is crucial for efficient scaling of the envisioned methodology.

## Figures and Tables

**Table 1:** Summary of the literature review on PINNs and related applications.

Author	Year	Method	Aim	Result
Chen et al. [26]	2025	Physics-informed encoder–decoder model	Predict carbon emissions and identify anomalies	Improved accuracy by 9.24% with enhanced robustness
Chen et al. [27]	2025	AI applications in sustainable energy	Review AI’s role in multi-energy systems	Identified challenges and proposed layered security strategies
Mittal et al. [28]	2025	Physics-informed neural network	Detect and classify wild animal activity	Achieved high accuracy and real-time alert generation
Pandiyan et al. [29]	2025	Physics-informed neural network (PINN)	Optimize electric water heater modeling	Enhanced computational efficiency and performance
Feng et al. [30]	2025	Uniform physics-informed neural network (UPINN)	Extract parameters for voltage stability	Improved accuracy in real-time voltage stability monitoring
Habib et al. [31]	2025	Block-based physics-informed neural network	Estimate inelastic response of base-isolated structures	Reduced computational cost and improved predictive performance
Nadal et al. [32]	2025	PINNs	Enhance simulation accuracy in power system dynamics	Improved predictive precision in power system simulations
Ventura Nadal et al. [33]	2025	PINNs	Improve power system simulation accuracy	Enhanced modeling and reduced computational error
Ko et al. [34]	2025	PINNs	Long-term prognostics of proton exchange membrane fuel cells	Achieved high accuracy in fuel cell lifespan prediction
Qin et al. [35]	2024	Inverse PINNs	Develop a digital twin-based approach for bearing fault diagnosis under imbalanced samples	Enhanced fault diagnosis accuracy and improved precision in cross-working-condition detection

**Table 2:** Summary of the literature review on blockchain, digital twin, and energy systems.

Author/Ref.	Methodology	Platform	Outcome	Challenge
Zahid et al. [55]	AI, Digital Twins, Blockchain, Metaverse	Smart Grid 3.0	Enhanced real-time monitoring, decentralized transactions, and system automation	Interoperability, scalability, cybersecurity, and data integrity issues
Sarker et al. [56]	Explainable AI (XAI) and Cybersecurity Modeling	DT Environments	Improved AI-driven cybersecurity automation and threat detection	Ensuring trustworthiness, human explainability, and AI transparency
Idrisov et al. [57]	ML and Digital Twin-Based Anomaly Detection	Power Electronics Dominated Grids (PEDGs)	Real-time tracking of power grid anomalies and cyberattack prevention	Handling complex grid operations and cybersecurity vulnerabilities
Meng et al. [58]	IoT, Blockchain, Cybersecurity	Smart Urban Energy Systems	Enhanced cybersecurity and efficient energy management in urban grids	Integration complexity and real-time cyber threat mitigation
Kavousi-Fard et al. [59]	DT for Renewable Energy Resources (RER)	Solar Energy Systems	Optimized energy management and real-time monitoring of solar grids	Variability in energy generation and reliability challenges
Kabir et al. [60]	IoT- DT Twin Systems	Smart Energy Grids	Improved operational efficiency, predictive maintenance, and grid sustainability	Addressing infrastructure compatibility and data security concerns
Cali et al. [61]	Cybersecurity, DT, AI	Energy Systems and Smart Cities	Enhanced efficiency, security, and sustainability in energy infrastructure	Ensuring secure real-time data transmission and system resilience
Jafari et al. [62]	Multi-Layer DT Model	Smart Grid, Transportation, and Smart Cities	Reliable energy distribution and improved grid operations	Managing real-time data flow and system scalability

**Table 3:** Renewable energy optimization comparison.

Baseline_Consumption_kWh	Optimized_Consumption_kWh	Solar_Output_kWh	Wind_Output_kWh	Total_Renewable_Output_kWh	Proposed_Model_Coverage_%	Traditional_Model_Coverage_%
7.617	7.537	0.075	0.011	0.086	1.124	0.915
7.47	7.03	0.19	0.269	0.459	6.14	5.287
14.063	12.984	0.146	1.123	1.269	9.024	7.688
7.495	6.772	0.12	0.662	0.782	10.436	7.626
7.719	6.881	0.031	0.885	0.917	11.874	8.666
12.594	12.097	0.031	0.482	0.514	4.079	3.074
9.497	8.937	0.012	0.56	0.571	6.017	4.647
12.767	11.799	0.173	1.033	1.207	9.451	7.388
5.654	5.517	0.12	0.026	0.146	2.591	2.166
9.876	9.601	0.142	0.198	0.339	3.437	2.445

**Table 4:** Comparison of predictive performance metrics.

Model	MAE	RMSE	R^2^	MBE	Accuracy	Precision	Recall	F1 Score
Proposed	0.2369	0.2980	0.9895	0.0296	0.9771	0.9783	0.9764	0.9774
Linear Regression	0.9971	1.2448	0.8182	−0.0139	0.9012	0.9204	0.8843	0.9052
Random Forest	0.6269	0.7859	0.9275	−0.0508	0.9446	0.9691	0.9235	0.9457
SVM	0.6973	0.8866	0.9077	−0.0267	0.9538	0.9552	0.9215	0.9381
LSTM	0.4987	0.6227	0.9545	−0.0226	0.9621	0.9626	0.9607	0.9617
XGBoost	0.5921	0.7394	0.9358	−0.0062	0.9503	0.9753	0.9313	0.9528

**Table 5:** Real-time performance metrics comparison.

Model	Response Time (s)	Energy Cost Reduction (%)	User Comfort Index (%)	Renewable Energy Utilization Rate (%)
**Proposed**	**0.5**	**35**	**96**	**40**
Linear Regression	2.5	15	80	20
Random Forest	1.8	25	90	30
SVM	2.0	22	85	22
LSTM	1.2	28	92	28
XGBoost	1.5	26	89	25

## Data Availability

Data is available and can be provided over emails querying directly to the corresponding author.
